# Resveratrol and Diabetic Cardiomyopathy: Focusing on the Protective Signaling Mechanisms

**DOI:** 10.1155/2020/7051845

**Published:** 2020-03-13

**Authors:** Yan-Jun Song, Chong-Bin Zhong, Wei Wu

**Affiliations:** ^1^Department of Cardiology, Peking Union Medical College Hospital, Peking Union Medical College and Chinese Academy of Medical Science, 1 Shuai Fu Yuan, Beijing 100730, China; ^2^Department of Cardiology, Heart Center, Zhujiang Hospital, Southern Medical University, Guangzhou, Guangdong 510282, China

## Abstract

Diabetic cardiomyopathy (DCM) is a common cardiovascular complication of diabetic mellitus that is characterized by diastolic disorder in the early stage and clinical heart failure in the later stage. Presently, DCM is considered one of the major causes of death in diabetic patients. Resveratrol (RSV), a naturally occurring stilbene, is widely reported as a cardioprotective substance in many heart diseases. Thus far, the specific roles of RSV in DCM prevention and treatment have attracted great attention. Here, we discuss the roles of RSV in DCM by focusing its downstream targets from both in vivo and in vitro studies. Among such targets, Sirtuins 1/3 and AMP-activated kinase have been identified as key mediators that induce cardioprotection during hyperglycemia. In addition, many other signaling molecules (e.g., forkhead box-O3a and extracellular regulated protein kinases) are also regulated in the presence of RSV and exert beneficial effects such as opposing oxidative stress, inflammation, and apoptosis in cardiomyocytes exposed to high-glucose conditions. The beneficial potential of an RSV/stem cell cotherapy is also reviewed as a promising therapeutic strategy for preventing the development of DCM.

## 1. Introduction

Diabetic cardiomyopathy (DCM), a chronic complication of diabetes first reported in 1972 [1], is one of the major causes of death in diabetic individuals [2, 3]. DCM is characterized by ventricular hypertrophy and fibrosis, which significantly increase the risk of subsequent clinical heart failure [4, 5]. Through cardiac ultrasound screening, it has been demonstrated that 37% of 101 diabetic patients without coronary artery disease and left ventricular hypertrophy exhibit significant subclinical left ventricular dysfunction [6]. It was found that the rate of myocardial dysfunction in 1093 type 1 diabetes (T1DM) patients was 15.5% [7], further indicating that myocardial dysfunction is common in diabetic patients. It was reported that the risk of heart failure is increased 5-fold in diabetic women and 2.4-fold in diabetic men compared with those without diabetes, and that is after adjustment for other risk factors such as age, hypertension, obesity, and dyslipidemia [8]. In addition, Nichols et al. reported that people with diabetes have a 2.5-fold increase in heart failure risk and are on average 5.5 years younger when they develop heart failure than are non-diabetic subjects [9].

For the mechanisms of DCM, impaired insulin metabolic signaling, hyperglycemia-induced abnormal AGE/receptor for advanced glycation end product (RAGE) signaling, mitochondrial dysfunction, increased fatty acid utilization, endoplasmic reticulum stress, and impaired calcium handling, in conjunction with coronary endothelial dysfunction, are considered pathogenic causes in type 2 diabetes- (T2DM-) induced DCM [5, 10, 11]. The molecular mechanisms of T1DM-induced DCM seem to overlap with changes in the hearts of patients with T2DM [12–14]. Notably, insulin deficiency may be the core factor contributing to T1DM-induced DCM, since insulin treatment can quickly reverse the phenotypes and the abnormalities observed in the hearts of patients with type 1 diabetes [15].

Resveratrol (RSV; 3,4′,5-trihydroxystilbene) has been shown to provide multiple beneficial effects in cardiovascular diseases such as heart failure [16, 17], myocardial ischemia/reperfusion (I/R) injury [18], and atherosclerosis [19]. The wide-ranging cardioprotective effects of RSV have attracted the attention of researchers in terms of its role against DCM. Currently, numerous observations indicate that RSV treatment is a promising therapeutic approach for disrupting the pathogenesis of DCM [20–22]. Regarding the mechanisms, Sirtuin 1 (Sirt1) is regarded as a primary cardioprotective downstream modulator of RSV in both T1DM and T2DM states, contributing to antioxidation, antiapoptosis, and calcium hemostasis improvement by further regulating its downstream molecules in cardiomyocytes [23–25]. In addition, RSV was revealed to regulate different signaling pathways in T1DM and T2DM. In the case of T1DM, adenosine monophosphate- (AMP-) activated kinase (AMPK) was suggested as one of the main targets of RSV, which activated several antioxidative and antiapoptotic mechanisms, thus preventing cardiac hypertrophy under HG conditions [26, 27]. Moreover, RSV was also reported to regulate several other molecules or receptors, such as Sirt3, NF-E2-related factor 2 (Nrf2), and RAGE, further magnifying its cardioprotective effect in T1DM conditions [28, 29]. For T2DM, RSV was revealed to alleviate cardiomyocyte inflammation, mitochondrial dysfunction, and metabolic disorders by downregulating tumor necrosis factor-*α* (TNF-*α*)/nuclear factor kappa B (NF-*κ*B) signaling and by preventing palmitoyl-CoA (P-CoA) respiratory sensitivity in cardiomyocytes [30, 31]. Taken together, RSV has been widely revealed as a cardiac protector in both T1DM and T2DM.

## 2. RSV: A Cardioprotective Substance

### 2.1. An Overview of RSV

RSV, a naturally occurring stilbene, is found in a variety of plant species, such as grapes and groundnuts [32]. RSV was first isolated from white hellebore in 1940 [33], and it was regarded as the potential explanation for the “French paradox” described in the 1990s [34]. In subsequent studies, RSV was found to be a polyphenolic cyclooxygenase inhibitor and a potential chemopreventive molecule that could be extracted from grape skin/seeds, red wine, and the root of *Polygonum cuspidatum* [16, 21].

**Figure 1:**
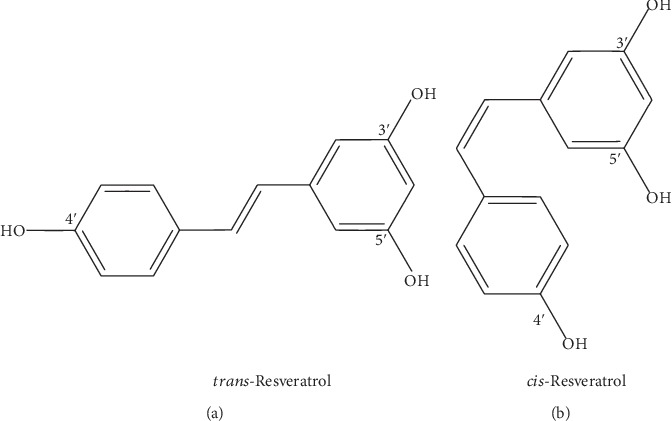
The structures of trans-RSV and cis-RSV.

The structure of RSV has been shown to be 3,4′,5-trihydroxystilbene, which exists as cis- and trans-isomers (Figure 1), and the latter is the most commonly found and stable form [35]. trans-RSV is determined to be primarily associated with health benefits [35, 36], and it is synthesized via the phenylpropanoid pathway [37]. A detailed analysis of the isomerization of geometric isomers in the case of RSV was recently presented by Wang and Chatterjee [36].

After oral administration, RSV is taken up at the apical membrane of erythrocytes by passive diffusion or through membrane transport [37]. In fact, resveratrol has high rates of oral absorption. It has been reported that approximately 70% of administered resveratrol can be absorbed [37, 38]. In the bloodstream, RSV can be found mainly in three different forms: glucuronide, sulfate, or free. The glucuronide- and sulfate-conjugated forms of resveratrol are the major metabolites of resveratrol, and they are formed in the intestine and liver [37, 38]. Free RSV can bind to albumin or lipoproteins, thus being transported in the bloodstream [38, 39]. The complexes of RSV and albumin or lipoproteins can be dissociated when the albumin or lipoproteins interact with the relative receptors at the cellular membrane, allowing RSV to be distributed into target organs, such as the liver and heart, and enter into cells [38–40].  Table 1 shows the information of some studies on the concentrations of the three forms of RSV in the plasma and heart [40–44].

### 2.2. The Cardioprotective Effects of RSV

The cardioprotective effects of RSV in cardiovascular diseases have been widely reported in numerous studies. As a well-known antioxidant, RSV-induced antioxidative properties in cardiomyocytes were shown to be vigorous contributors to cardioprotection. Mechanistically, NADPH oxidase suppression, the reduction of reactive oxygen species (ROS) generation, and preservation of critical antioxidant enzyme activity (e.g., superoxide dismutase (SOD), catalase, and glutathione peroxidase) were shown to be the main effects of RSV. These RSV-induced alterations consequentially reduced lipid peroxidation, increased cardiomyocyte viability, and alleviated cardiac hypertrophy [45, 46]. Moreover, recent studies also found that RSV significantly alleviated cardiac metabolic disorders such as recovering glucose homeostasis, normalizing free fatty acid oxidation (FFAO), and enhancing utilization glucose, which optimized cardiac energy metabolism, especially in cardiomyocytes in HG conditions [47–49]. Besides, the inhibitory effects of RSV on inflammation, apoptosis, and serum cholesterol concentration were also reported [7, 23]. These effects further contribute to cardioprotection in many cardiovascular diseases, such as myocardial infarction, myocardial I/R injury, and DCM [23].

In addition to cardiomyocytes, endothelial cell is another target for RSV conferring protective effects in cardiovascular diseases. Presently, several studies have suggested that RSV preserves endothelial mitochondrial oxidation, maintains endothelial function, induces vasodilation and vascular angiogenesis, which subsequently improves blood flow and cardiac function [46, 50]. As for the mechanisms, the activation of the endothelial nitric oxide synthase (eNOS)/nitric oxide (NO) axis was suggested to be the primary target. For instance, recent studies suggested that RSV increased NOS expression and prevented eNOS uncoupling in endothelial cells, which improved endothelial angiogenesis and coronary flow, hence inducing cardioprotection in rat I/R hearts [51–55]. RSV-induced activation of the eNOS/NO axis and similar endothelial protective effects have also been widely reported in other cardiovascular diseases [56–59]. Additionally, several proangiogenic factors, such as vascular endothelial growth factor (VEGF) and heme oxygenase-1, were also determined to be induced by RSV, which contributed to the preservation of endothelial cells in heart diseases [60–62].

## 3. RSV: Cardioprotective Signaling against the Development of Diabetic Cardiomyopathy

**Figure 2:**
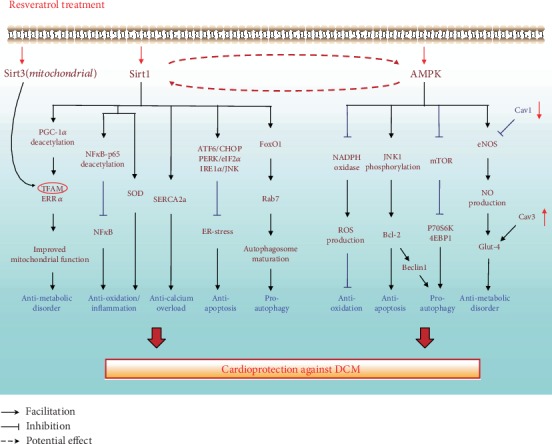
The involvements of SIRT1/3 and AMPK in the RSV-induced cardioprotection against the development of DCM. SIRT1 and AMPK are the key downstream molecules of RSV, mediating various molecules and subsequently exerting cardioprotective effects such as antioxidative, antiapoptosis, and proautophagy in cardiomyocytes in high-glucose condition. Moreover, Sirt3, a mitochondrial Sirt member, is also shown to coregulate TFAM with SIRT1 and together contribute to a antimetabolic disorder effect eventually. Additionally, the relationship between RSV and Cav1/3 is also described in this picture.

RSV has been recently reported to exert cardioprotective effects in diabetes, such as inhibiting oxidative stress [47, 63], alleviating the inflammatory response [64], reducing apoptosis [22], attenuating impaired autophagy [65], improving calcium homeostasis [66], and alleviating metabolic disorders [67]. These cardioprotective effects are attributed to its downstream signaling mechanisms. Among these factors, sirtuin members (e.g., Sirt1 [23] and Sirt3 [68]) and AMPK [26] are noticeable downstream molecules of RSV, further triggering various signaling pathways against DCM injury (Figure 2).

**Figure 3:**
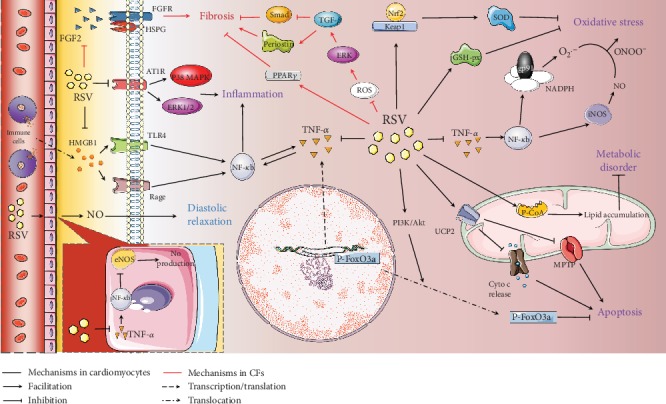
Other signaling mechanisms regulated by RSV in a DCM heart. In high-glucose condition, RSV-induced cardioprotection can be divided into 6 parts: (1) RSV inhibits the TNF-*α*/NF-*κ*B axis, subsequently suppressing iNOS and NADPH in cardiomyocytes, which finally decreases the ONOO^−^ levels and the following oxidative stress. Moreover, the RSV-induced upregulated GSH-px and Nrf-2 also contribute to the antioxidative stress. (2) RSV also inhibits the TNF-*α*/NF-*κ*B axis in vascular endothelial cells, thereby enhancing NO release and improving cardiac diastolic function. (3) RSV inhibits AT1R/MAPK and HMGB1/TLR4/RAGE pathways, thus conferring the anti-inflammatory effects in cardiomyocytes during hyperglycemia. (4) As for the antiapoptotic mechanisms, RSV activates the PI3K/Akt pathway and UCP2, which consequentially promotes the cytoplasm translocation of p-FoxO3a and inhibits the Cyto c release and MPTP opening, respectively. (5) Furthermore, RSV exerts antimetabolic effects via activating p-CoA and lipid accumulation in mitochondria. (6) In mCFs, RSV exerts antifibrotic effects via inhibiting the ROS/ERK/TGF-*β* pathway and activating PPAR*γ*. Besides, RSV inhibits the FGF2 binding activity to FGFR and HSPG in mCF surface, which also reduces mCF proliferation in high-glucose condition.

Besides, RSV also regulates numerous other downstream cardioprotective signaling mechanisms, such as high mobility group box 1- (HMGB1-) dependent and mitogen-activated protein kinase- (MAPK-) dependent pathways, which further block DCM development [29, 69]. In this review, we elucidate these mechanisms by dividing them into five sections (antioxidative, anti-inflammatory, antimetabolic disorder, antiapoptosis, and antifibrotic signaling) according to their main effects in the hearts with DCM upon RSV treatment (Figure 3). Detailed information on the studies revealing these protective signaling mechanisms is summarized in Table 2.

### 3.1. RSV and Sirtuin 1

Sirts are a family of conserved nicotinamide adenine dinucleotide- (NAD^+^-) dependent histone deacetylases [70]. The Sirt family contains 7 members (Sirt1-7), each of which has been widely implicated in the regulation of many physiological processes such as energy metabolism and cellular senescence [71–73]. In a high-glucose (HG) state, the expression of different Sirts is significantly altered. The expression and activity of Sirt1, Sirt2, Sirt3, and Sirt5 is reduced in a streptozotocin- (STZ-) induced type 1 diabetes mellitus (T1DM) model, while the level of Sirt3 is increased in a high-fructose diet-induced T2DM model [74]. These mechanisms further induce cardiac fibrosis and dysfunction in diabetes. RSV administration has been observed to prevent these alterations in DCM rat hearts, thus providing significant cardioprotection [74]. In particular, Sirt1 and Sirt3 have been suggested to be the main downstream mediators of RSV-induced cardioprotective effects against DCM injury [23, 68].

Sirt1, the first Sirt family member to be discovered, is found in the nucleus of cardiomyocytes, and it acts as a cardioprotective mediator in the hearts with DCM. Researchers have shown that Sirt1 can interrupt the progression of DCM by inhibiting oxidative stress, calcium overload, metabolic disorders, and apoptosis by mediating several molecular factors, such as activating peroxisome proliferator-activated receptor gamma-coactivator alpha (PGC-1) and sarcoplasmic reticulum calcium ATPase (SERCA2a) [75].

The roles of Sirt1 involved in RSV-induced cardioprotection in DCM have been further explored by several recent studies [23, 76]. Presently, scholars demonstrated that treatment with RSV (25 mg/kg/d for 5 days) significantly reversed the HG-induced reduction in Sirt1 expression and alleviated cardiac dysfunction in mice with DCM, indicating that Sirt1 serves as a downstream cardioprotective target of RSV in cardiomyocytes [23]. It was suggested that the mechanism of Sirt1 activation by RSV could be attributed to improved levels of NAD^+^ [24]. In addition, the inhibition of miR-34a has been indicated to be a potential mechanism. In the setting of cardiomyocyte hypoxia/reoxygenation injury, RSV treatment was shown to significantly suppress the miR-34a upregulation and consequently induced Sirt1 expression [77]. This mechanism was also observed in cardiomyocytes during hyperglycemic conditions. In 2018, investigators found that therapeutic inhibition of miR-34a restored Sirt1 expression and subsequently reduced HG-induced cardiomyocyte apoptosis [78]. Taken together, the miR-34a/Sirt1 axis is supposed to be regulated by RSV in cardiomyocytes during hyperglycemia. Hence, further studies should focus on the concrete association between RSV treatment and the miR-34a/Sirt1 axis in DCM hearts.

For the subsequent cardioprotective effects of Sirt1 in the presence of RSV, improving metabolic disorders is shown to be the key part. Specifically, activation of Sirt1 by RSV treatment induced positive regulation of mitochondrial biogenesis and function via PGC-1*α* deacetylation, which augmented the expression of mitochondrial regulatory genes such as estrogen-related receptor *α* and mitochondrial transcription factor (TFAM) [23, 25]. Since disorders of glucose and lipid metabolism are known to be closely related to mitochondrial dysfunction in the development of DCM, these protective effects on mitochondrial function induced by Sirt1 lead to improvements in metabolic disorders, such as lowering blood glucose and reducing insulin resistance, thereby preserving cardiac function under diabetic conditions [23].

Sirt1 is further reported to inhibit oxidative stress in cardiomyocytes under HG conditions upon RSV treatment [25]. Using a high-fructose diet in vivo model, researchers found that activation of Sirt1 deacetylated NF-*κ*B-p65 at lysine 310 and histone 3 at lysine 9, which subsequently decreased NF-*κ*B-p65 activity and alleviated oxidative stress in diabetic myocardial tissue [24]. Inhibition of Sirt1 in HG-cultured H9C2 cells was shown to decrease this antioxidative effect, suggesting that the RSV/Sirt1/NF-*κ*B-p65 pathway inhibited oxidative stress in cardiomyocytes exposed to HG [24]. Moreover, according to an in vivo HG rat model, RSV treatment (50 mg/kg/d for 16 weeks) upregulated cardiac superoxide dismutase (SOD) in myocardial tissue and alleviated cardiac oxidative stress, ultimately attenuating cardiac hypertrophy in the hearts with DCM [25]. These changes were abolished by treatment with sirtinol (a Sirt1 inhibitor), further showing that RSV induced suppression of oxidative damage via by enhancing SOD activity in a Sirt1-dependent manner [25].

Despite the mechanisms described above, the improvement of calcium homeostasis in cardiomyocytes is shown as another cardioprotective role of Sirt1. In this regard, investigators suggested that RSV alleviated calcium overload in cardiomyocytes in a HG state by enhancing SERCA2a activation in a Sirt1-dependent manner [66]. Regarding the mechanisms underlying Sirt1 activation of SERCA2a, specificity protein 1 (Sp1) was suggested to be the potential target, as one study found multiple binding sites for the Sp1 receptor found within the glucose-responsive region [66], and Sp1-induced upregulation of SERCA2 was demonstrated in Sol8 muscle cells in another study [79]. However, the association of Sp1 with Sirt1 in alleviating calcium overload in cardiomyocytes has not been well elucidated thus far. Therefore, whether Sp1 is involved in Sirt1-induced SERCA2a activation in DCM hearts should be further studied.

The antiapoptotic effect induced by RSV-activated Sirt1 has also been reported in cardiomyocytes exposed to HG conditions [80]. Using HG-cultured H9C2 cells, researchers found that RSV supplementation (10 *μ*M) reduced cardiomyocyte apoptosis by suppressing endoplasmic reticulum (ER) stress [80]. Mechanistically, the induction of Sirt1 was observed to reduce the expression of activating transcription factor 6 (ATF6), p50, and anti-CCAAT/enhancer-binding protein homologous protein (CHOP), which thereby suppressed the levels of phosphorylated protein kinase-like endoplasmic reticulum kinase (PERK) and eukaryotic initiation factor 2*α* (eIF2*α*, the downstream molecule of ATF6 and PERK) [80]. The levels of phosphorylated inositol-requiring transmembrane kinase and endonuclease 1*α* (IRE1*α*) and phosphorylated Jun-amino-terminal kinase 1/2 (JNK 1/2) were also found to be decreased by RSV in this study [80]. These mechanisms finally contributed to the inhibition of ER stress and increased cardiomyocyte survival rates. Silencing Sirt1 inhibited these antiapoptotic effects, again supporting the idea that activated Sirt1 contributed to attenuated ER stress and subsequent cardiomyocyte apoptosis by downregulating these signaling pathways during hyperglycemic conditions [80].

Furthermore, roles for Sirt1 in RSV-regulated autophagy have also been identified [65]. In this respect, investigators have suggested that RSV treatment (60 mg/kg/day for 4 weeks) increased Sirt1 activity, thus ameliorating insufficient myocardial autophagic flux in STZ-induced diabetic mice [65]. In terms of specific mechanisms, activated Sirt1 wash shown to increase the DNA-binding ability of forkhead box-O1 (FoxO1) at the promoter region of Rab7 and subsequently enhanced Rab7 expression, which contributed to the maturation of autophagosomes and their fusion with lysosomes, thus eventually causing autophagic flux improvement in the hearts with DCM [65, 81]. Moreover, knocking down Sirt1 with siRNA suppressed the RSV-induced upregulation of FoXO1 and Rab7, as well as the amelioration of HG-impaired autophagic flux, while Rab7 knockdown only abolished the cardioprotective effects but did not affect the protein levels of Sirt1 or FoXO1 [65]. This result supports the notion that RSV improves autophagic flux through the Sirt1/FoXO1/Rab7 pathway, which provides subsequent cardioprotection in diabetes [65]. Thus far, Sirt1 is determined to be one of the key mediators contributing to RSV-induced cardioprotective effects, including attenuating oxidative stress, inhibiting cardiomyocyte apoptosis, decreasing calcium overload, and ameliorating autophagic flux under HG conditions.

### 3.2. RSV and Sirtuin 3

As stated above, Sirt3 is another cardioprotective downstream mediator in RSV-induced cardioprotection against DCM injury [82, 83]. Sirt3 is a major deacetylase in the mitochondrial matrix, and it plays important roles in regulating mitochondrial energetics and cellular redox status [84, 85]. In T1DM, HG-induced Sirt3 reduction decreased the activity of the mitochondrial electron transport chain complex and the subsequent production of ATP in cardiomyocytes, which resulted in deficient mitochondrial energetics and reduced cardiac size [68]. Recently, several studies have determined that overexpressing Sirt3 through preconditioning or genetic technology was able to activate Parkin-dependent mitophagy, which eliminated superfluous and damaged mitochondria in cardiomyocytes during diabetes [82, 86].

Regarding the role of Sirt3 in the cardioprotective effects of RSV against DCM injury, investigators have shown that RSV (25 mg/kg/day) significantly increases the expression and activity of both Sirt1 and Sirt3 and subsequently enhances mitochondrial function and biogenesis in diabetic myocardial tissues [68]. Mitochondrial TFAM was considered to be one of the key molecular downstream targets of Sirt3. In this study, TFAM was observed to be significantly activated, and attenuated the dysregulation of the ETC complex, which increased the levels of ATP and mitochondrial content in cardiomyocytes [68]. These changes ameliorated cardiomyocyte apoptosis, cardiac atrophy, and fibrosis in diabetes [68]. However, TFAM activation and these subsequent protective effects were accompanied by increased activity of both Sirt1 and Sirt3. To explore the precise roles of Sirt3, investigators silenced Sirt1 and/or Sirt3 in H9C2 cells [68]. Interestingly, the enhanced activity of TFAM was significantly reduced in Sirt3-silenced cells but not in Sirt1-silenced cells. Consistently, silencing Sirt3 has also been shown to enhance the acetylation of TFAM, which was not observed in Sirt1-silenced cells [68]. These results suggest that Sirt3 is the key mediator in regulating the activity and acetylation status of TFAM in cardiomyocytes exposed to HG conditions [68]. Paradoxically, it was reported that Sirt1 also induced TFAM and its subsequent cardioprotective effects against DCM injury [23]. To resolve this discrepancy, we suggest that Sirt3 may be activated through two pathways upon RSV treatment: (1) Sirt3 is activated by RSV directly, and (2) Sirt3 is activated downstream of RSV-induced Sirt1, which is responsible for activating TFAM. To be noticed, the second hypothesis was identified during myocardial I/R injury. In the setting of myocardial I/R model, Lochner et al. found that melatonin-induced Sirt1 activated Sirt3 and consequently increased TFAM activity, thus exerting cardioprotective effects against I/R injury [87]. Therefore, whether the Sirt1/Sirt3/TFAM axis exists in an RSV-treated heart with DCM is worthy of further investigation in future studies.

### 3.3. RSV and AMPK Signaling

AMPK is a serine-threonine kinase that regulates cellular metabolism by mediating glucose homoeostasis [88]. In recent studies, AMPK was suggested to be a key therapeutic modulator mediating several signaling pathways and exerting protective effects against the development of DCM upon RSV treatment [27, 89]. Among the effects, antioxidation is a key result of AMPK signaling. It has been reported that RSV administration (50 *μ*M) increased AMPK activity and subsequently downregulated NADPH oxidase, a major source of ROS generation [90], which alleviated oxidative damage in neonatalrat cardiomyocytes exposed to HG insult [26]. Concomitantly, activated AMPK was also shown to alleviate the reduction of cardiac antioxidant enzyme activities such as SOD, catalase, and GSH-px, further reducing oxidative stress in cardiomyocytes exposed to HG conditions [26]. Besides, AMPK was also involved in RSV-induced antiapoptotic effects in DCM hearts via increasing Bcl-2 levels and reducing Bax levels [26, 91]. Mechanistically, researchers found that RSV (25 *μ*M)- activated AMPK increased the phosphorylation of JNK1, further augmenting Bcl-2 phosphorylation and reducing cardiomyocyte apoptosis [91]. Interestingly, this change was observed to not only induce antiapoptotic effects but also promote cardiomyocyte autophagy during hyperglycemia [91]. Specifically, the phosphorylation of Bcl-2 further promoted the dissociation of Beclin1 from Bcl-2, which induced cardiomyocyte autophagy and cardioprotection concomitantly in diabetic conditions. Moreover, this study further suggested that the suppression of mammalian target of rapamycin (mTOR) activation and its downstream components (P70S6K and 4EBP1) was also induced by AMPK, which also contributed to cardiomyocyte autophagy under diabetic conditions upon RSV treatment [91–93]. The protective effects described above are abolished by compound C, a pharmacologic inhibitor of AMPK, again supporting that AMPK serves as a key factor in the RSV-induced antioxidative, antiapoptotic, and proautophagic actions in cardiomyocytes during diabetes [26, 91].

In addition to the protective effects described above, RSV-induced AMPK was also suggested to confer an antimetabolic effect through enhanced Glut-4 expression on the surface of cardiomyocytes in diabetic conditions. For the specific mechanism involved, an in-depth study showed that RSV treatment (65 mg/kg) activated AMPK and consequently phosphorylated eNOS on Ser1177, contributing to NO production, which increased Glut-4 translocation to the cell surface [94] and glucose uptake during hyperglycemia [67]. Moreover, this study also reported that RSV decreased the expression of caveolin-1 (Cav-1), a physiological inhibitor of eNOS in the endothelial plasma membrane microdomain caveolae [95]. As a result, more eNOS was released into the cytosol and phosphorylated by AMPK, which further improved NO production and enhanced cellular glucose uptake during hyperglycemia. Besides, researchers have further documented that the AMPK/Akt pathway is highly activated by RSV treatment (0.1 mg/kg) in STZ-induced T1DM hearts, also resulting in increased levels of membranous Glut-4 and enhanced glucose uptake [96, 97]. This result indicates that the RSV-activated AMPK/Akt signaling pathway is another contributor to the increase in Glut-4 translocation and the subsequent protective actions observed regarding metabolic hemostasis.

Sirt1 activation may be involved in the cardioprotective actions of AMPK, as AMPK and Sirt1 were reported to regulate each other and share common target molecules in the maintenance of cellular energy metabolism in cardiomyocytes exposed to HG conditions [98, 99]. A previous study showed that AMPK increased NAD^+^ levels and activated Sirt1, leading to an increase in the AMP/adenosine triphosphate (ATP) ratio and enhancing the binding activity of AMP to the regulatory *γ* subunit. The increased binding activity was shown to induce a conformational change in the AMPK complex and attenuate its dephosphorylation ability, which in turn resulted in protective effects in cardiomyocytes exposed to hyperglycemic conditions [98]. Moreover, the interaction between AMPK and Sirt1 was also investigated in other heart diseases [100]. In a rat model subjected to myocardial I/R injury, AMPK was demonstrated to increase cellular NAD^+^ levels and the NAD/NADH ratio, which enhanced Sirt1 activity [100]. Activated Sirt1 was shown to deacetylate liver kinase B1, which is a key upstream activator of AMPK, and to upregulate AMPK activity in turn; further, Sirt1 induced cardioprotection against I/R injury [100]. Given that AMPK and Sirt1 are both involved in the beneficial actions of RSV against DCM injury, it is worth further investigating whether the interaction between AMPK and Sirt1 is also mediated by RSV in cardiomyocytes during HG conditions.

### 3.4. RSV and Other Signaling Mechanisms

#### 3.4.1. Antioxidative Stress Signaling

Of the RSV-induced antioxidative targets, Nrf2, an indispensable antioxidative mediator [101], is a key molecule augmenting antioxidative enzyme activity and alleviating oxidative stress in cardiomyocytes exposed to HG conditions [28, 102]. For the RSV-induced mechanisms increasing Nrf2 expression in the hearts with DCM, upregulated Sirt1 has been suggested to be one of the major activators [23]. Additionally, Kelch-like erythroid cell-derived protein with CNC homology-associated protein 1 (Keap-1) suppressed Nrf-2 under basal conditions through its ubiquitination-proteasomal degradation [103], and Keap-1 was indicated to be downregulated by RSV, leading to the subsequent activation of Nrf-2. Specifically, by using an obese asthma rat model, investigators demonstrated that RSV decreased Keap-1 expression and augmented Nrf2 levels. This result consequentially increased antioxidant enzyme activities (e.g., GSH and SOD), thus decreasing ROS production and oxidative stress in cardiomyocytes [104]. Given that Keap-1 is also increased in the cardiomyocytes exposed to HG [105], it is reasonable to speculate that the antioxidative effects of RSV rely on the Keap-1/Nrf2 signaling pathway, which is worthy of further confirmation. For the subsequent cardioprotective mechanisms, it was suggested that the Nrf2/antioxidant response element (ARE) antioxidant system played a key role in the antioxidative capacity of Nrf2 against DCM injury [106]. Specifically, RSV-induced Nrf2 upregulation promoted the downstream expression of antioxidative Nrf2 targets, such as SOD1 and SOD2, which inhibited ROS accumulation and oxidative stress in cardiomyocytes during hyperglycemic conditions [28].

In addition, the inhibition of the TNF-*α*/NF-*κ*B pathway was also involved in RSV-induced antioxidative effects observed in cardiomyocytes facing hyperglycemic conditions [30]. Using a HG rat model, investigators found that RSV (20 mg/kg/d for 4 weeks) attenuated TNF-*α* expression and inhibited NF-*κ*B-*α* phosphorylation, which subsequently suppressed NF-*κ*B activity in myocardial tissue. Decreased NF-*κ*B activity inhibited the expression of NADPH oxidase and O_2_^·-^ levels, thus protecting cardiomyocytes from oxidative stress in diabetic conditions [30]. Moreover, RSV also reduced inducible nitric oxide synthase (iNOS) in an inhibitory NF-*κ*B-dependent manner [30]. As iNOS-induced pathological concentrations of NO were demonstrated to result in nitrative stress by generating the nitrative molecule peroxynitrite (ONOO^−^) with O_2_^·-^ [107], the inhibition of iNOS by RSV could reduce cardiac oxidative/nitrative stress during HG conditions [30, 108]. Besides, eNOS expression in the coronary endothelium would be increased by RSV-mediated NF-*κ*B inhibition and subsequently increased NO production and release, which finally improves cardiac diastolic function [30, 109]. The present study further demonstrated that recombinant TNF-*α* reversed the cardioprotective effects observed above, again demonstrating that inhibition of TNF-*α*/NF-*κ*B signaling served as a downstream axis by which RSV mediated antioxidative effects in cardiomyocytes exposed to HG [30].

#### 3.4.2. Anti-Inflammatory Signaling

Signaling from the MAPK family has been reported to contribute to the pathogenesis of cardiac hypertrophy via exacerbating inflammation in diabetic conditions [110]. Angiotensin type 1 receptor (AT1R) is thought to induce cardiac dysfunction by activating its two major downstream MAPK signaling members (extracellular signal-regulated kinases (ERK) 1/2 and p38 MAPK) under HG conditions [111, 112]. Notably, inhibition of AT1R-ERK/p38 MAPK signaling is involved in the anti-inflammatory actions of RSV in cardiomyocytes during diabetic conditions [69]. In a recent study, RSV administration (80 mg/kg/d) significantly suppressed AT1R, ERK1/2, and p38 MAPK phosphorylation in a STZ-induced T1DM model. This result was shown to further attenuate the inflammatory response and improve cardiac function in the hearts with DCM, indicating the anti-inflammatory roles of AT1R-ERK/p38 MAPK signaling inhibition under RSV treatment.

HMGB1, a proinflammatory cytokine secreted from immune cells into serum [113], was significantly induced by HG conditions, followed by increased NF-*κ*B transcriptional activity and sustained upregulation of TNF-*α* and interleukin-6 (IL-6) expression in cardiomyocytes [29]. HMGB1 blockage was reported to be reduced by RSV during hyperglycemia and further protected heart tissue from DCM injury. Specifically, RSV treatment (5 or 25 mg/kg/d for 5 months) led to suppressed HMGB1 levels in both serum and hearts, together with the alleviated inflammatory responses in the STZ-induced diabetic hearts [29]. Moreover, researchers further investigated the underlying mechanism and reported that the decreased HMGB1 levels subsequently downregulated RAGE and toll-like receptor 4 (TLR4), in conjunction with their downstream proinflammatory cytokine NF-*κ*B, eventually inducing inflammatory response mitigation [29]. This result indicates that an RSV/HMGB1-RAGE/TLR4-NF-*κ*B signaling pathway exists and contributes to ameliorating the inflammatory response in diabetic hearts. Besides, HMGB1 was reported to activate E26 transformation-specific sequence-1 (Ets-1) via ERK1/2 activation and subsequently exacerbate the inflammatory response in HG conditions [114, 115]. Considering that ERK1/2 has been shown to be reduced by RSV administration, as stated previously, it is worth discussing whether RSV can regulate the inflammatory response in the DCM hearts through the HMGB1/ERK/Ets-1 pathway.

#### 3.4.3. Antiglucose/Fat Metabolic Disorder Signaling

In the hearts with DCM, cardiomyocyte fat metabolic disorder plays a key role in exacerbating cardiac function. HG-induced FA uptake/oxidation was one of the key contributors to fat metabolic disorders, as it was shown to aggravate mitochondrial dysfunction and glucose uptake reduction in myocardial tissue [116, 117]. In this regard, investigators found that treatment with RSV decreased free FA serum levels and cardiac FAO, which was associated with inhibited acetyl-CoA, improved pyruvate dehydrogenase activity, and glucose oxidation in the myocardium of diabetic animals [47]. Moreover, HG-induced P-CoA sensitivity reduction was also shown to disturb fat metabolic hemostasis, as it resulted in lipid accumulation within the heart tissue in diabetes [118–120]. Restoration of P-CoA sensitivity was shown to be involved in the beneficial actions of RSV against cardiomyocyte fat metabolic disorder in HG conditions [31]. Using a Zucker diabetic fatty rat model, scholars reported that RSV (200 mg/kg/d for 6 weeks) increased cardiac P-CoA sensitivity and subsequently normalized reactive lipid accumulation in mitochondria, which ultimately alleviated cardiac dysfunction [31]. Despite fat metabolism, glucose metabolic abnormalities are another key player in diabetes-stimulated cardiac dysfunction [121], which is mainly attributed to mitochondrial dysfunction and insulin resistance [122–124]. Fat metabolic disorder plays an important role in mitochondrial dysfunction, as researchers found that FAO and P-CoA sensitivity reduction compromised mitochondrial respiratory function and led to elevated ROS production by the mitochondria in the myocardium during hyperglycemic conditions [31, 125]. Therefore, the mechanisms by which RSV reduces fat metabolic disorders stated above concomitantly alleviated mitochondrial oxidative phosphorylation and increased ADP sensitivity, further improving cardiomyocyte energy metabolism and glucose uptake under HG conditions. In addition to mitochondrial protection, RSV was also suggested to increase glucose uptake by alleviating cardiac insulin resistance in a Glut-4-dependent manner. For the mechanisms involved, except the activated AMPK/Akt axis discussed earlier, RSV-induced Cav-3 enhancement was another mechanism triggering cardiac membrane Glut-4 translocation in diabetes, linked with the increased internalization of glucose in the cardiomyocytes in diabetes [67]. Taken together, these results indicated that RSV attenuated fat and glucose metabolic abnormalities, thereby protecting against metabolic shifting in cardiac tissue under HG conditions.

#### 3.4.4. Antiapoptotic Signaling

PI3K/Akt signaling has been widely considered to be antiapoptotic pathway in cardiomyocytes exposed to HG insult [126, 127]. Presently, the role of the RSV-regulated PI3K/Akt pathway in DCM has caused concerns [128]. In a recent study, the PI3K/Akt pathway was observed to be activated by treatment with RSV (5 mg/kg/day for 8 weeks), and it also augmented the survival rates of cardiomyocytes cultured in hyperglycemic conditions [128]. PI3K/Akt signaling activation was followed by an enhancement in p-FoxO3a translocation to the cytoplasm in cardiomyocytes under HG conditions, which suppressed cardiomyocyte apoptosis by inhibiting Bim and Fas-L [128, 129]. This result was abrogated by treatment with LY294002, an inhibitor of the PI3K/Akt pathway, documenting that PI3k/Akt/FoxO3a was involved in the antiapoptotic effect of RSV in the DCM hearts [128]. Furthermore, Ni et al. found that sustained treatment with RSV (20 *μ*M) induced a modest increase in FoxO1 and FoxO3, which phosphorylated Akt in turn, indicating the existence of feedback regulation in the axis [130]. Notably, researchers also reported that RSV inhibited the PI3K/Akt signaling pathway and subsequently exerted proapoptotic effects in cardiac fibroblasts (CFs) exposed to HG conditions [131]. This result indicated that RSV concomitantly induced CF apoptosis, which further reduced the development of cardiac fibrosis in hyperglycemic conditions.

In addition, another antiapoptotic substance, uncoupling protein 2 (UCP2), a proton transporter in the mitochondrial inner membrane, was also reported to be involved in RSV-induced cardioprotection during hyperglycemia [132, 133]. Generally, the cardioprotective effects of UCP2 in DCM are attributed to the inhibition of mitochondrial ROS and better utilization of free fatty acid substrates, which eventually protect against oxidative stress and ameliorates cardiac dysfunction [134]. For its specific roles in the DCM hearts upon RSV treatment, UCP2 activation was suggested to relieve diabetic myocardial fibrosis and myocardial apoptosis [135]. Mechanistically, investigators determined that RSV-mediated activation of UCP2 decreased the mitochondrial permeability transition pore opening level and suppressed cytochrome c release from mitochondria in cardiomyocytes under HG conditions [135]. These effects consequently reduced cardiomyocyte apoptosis and cardiac hypertrophy in DCM hearts [135]. Moreover, knocking down UCP2 abrogated this antiapoptotic effect, again demonstrating that UCP2 was a downstream antiapoptotic target of RSV in DCM hearts.

#### 3.4.5. Antifibrotic Signaling

Transforming growth factor-*β* (TGF-*β*) is a profibrotic cytokine that induces the production of extracellular matrix proteins in the hearts [136]. In diabetic conditions, two TGF-*β*-related profibrotic axes, the ROS/ERK/TGF-*β*/periostin and TGF-*β*/Smad3 pathways, were significantly suppressed by RSV [137]. Specifically, investigators reported that RSV inhibited ROS production and ERK activity, which significantly suppressed HG-induced proliferation of primary mouse CFs (mCFs) [137]. Moreover, the suppression of ERK further normalized the expression of TGF-*β* and consequently abolished periostin overexpression, thus attenuating the differentiation of mCFs under HG conditions [137]. Additionally, TGF-*β*1/Smad3 was inhibited by RSV both in vivo and in vitro [137]. In one study, investigators suggested that RSV treatment (10 mg/kg/day) reversed the HG-induced upregulation of TGF-*β*1 and subsequently suppressed Smad-3 phosphorylation, which eventually alleviated cardiac fibrosis in diabetic conditions [137]. Taken together, these results indicate that RSV inhibits the ROS/ERK/TGF-*β*/periostin and TGF-*β*1/Smad3 pathways, significantly alleviating myocardial fibrosis during hyperglycemic conditions.

RSV also regulates fibroblast growth factor 2 (FGF2), a paracrine molecule that contributes to cardiac fibrosis by binding to its receptors, such as FGFR1 and heparan sulfate proteoglycans (HSPGs), on the surface of mCFs; from there, it subsequently exerts its antifibrotic ability in diabetes [138–140]. Using the STZ and nicotinamide-induced T2DM model, researchers showed that RSV treatment (22.04 mg/kg/d for 6 weeks) downregulated the expression of FGF2 and HSPGs (e.g., glypican-1 and syndecan-4), which was associated with the alleviation of cardiac fibrosis and the improvement of cardiac dysfunction [140]. In addition, RSV was reported to inhibit peroxisome proliferator-activated receptor gamma (PPAR*γ*), further attenuating cardiac fibrosis in HG conditions [140]. Regarding the mechanism involved, Sirt1 was involved in the inhibition of PPAR*γ* induced by RSV [141]. Under high-fat conditions, RSV treatment was shown to activate Sirt1, which subsequently interacted with CDK2-associated cullin 1 (CACUL1), thereby repressing PPAR*γ* and adipogenesis [141]. Since high-fat conditions have been shown to be involved in the T2DM model in many studies, whether the Sirt1/CACUL1/PPAR*γ* axis is also modulated by RSV treatment in hearts with DCM needs further investigation.

## 4. RSV and Stem Cells

Stem cell therapy, such as the kinds involving mesenchymal stem cells (MSCs), represents a promising treatment strategy for cardiovascular diseases [142, 143]. Previous in vivo studies have reported that RSV augmented the proliferation and survival of stem cells in conjunction with cardioprotection in several pathological conditions, including hyperglycemia [22, 144]. Recently, the cardiac therapeutic potential of combined treatment of RSV and MSCs for DCM has attracted great attention from researchers.

Investigators have reported that RSV pretreatment (0.1 *μ*mol for 7 days) increases the proliferative capacity and antioxidant properties of MSCs in vitro [145]. Furthermore, in STZ-induced diabetic rats, systemic RSV treatment (2.5 mg/kg/d for 8 weeks) and RSV-preconditioned MSC cotherapy also showed increased antiapoptotic and antifibrotic capacities in the hearts with DCM compared with those of the other groups (RSV group, RSV-treated MSC group, RSV combined with non-RSV pretreated MSC group, and control group) [145]. Mechanistically, the combined treatment was suggested to minimize the area (%) for cardiac immunostaining of secreted frizzled-related protein (sFRP2) [145]. Since sFRP2 was shown to activate Wnt/*β*-catenin signaling in cardiac fibroblasts and contribute to cardiac extracellular matrix remodeling [146, 147], the strongest inhibitory effect of cotreatment on the expression of sFRP2 was shown to inhibit the Wnt3a/*β*-catenin pathway in CFs exposed to HG conditions, which was superior in ameliorating fibrosis in cases of DCM [145]. Collectively, both RSV pretreatment and RSV-combined treatments enhance the cardioprotective properties of MSCs, indicating that RSV-MSC therapy is a promising therapeutic approach for treating DCM. Therefore, further investigation is suggested to determine whether similar cardioprotective results could be observed in broader animal trials or clinical trials.

## 5. Doses and Duration of RSV for DCM

Although RSV exhibits great cardioprotective features in the diabetic state, as shown in the sections above, its health benefits, however, appear to be dose dependent [148, 149]. For DCM, previous data showed that an oral dosage of 5 mg/kg/day or an intraperitoneal injection with 1 mg/kg/day was the minimum effective dose for cardioprotection [64], and a higher dose was observed to bring more effective and efficient cardioprotective effects on glycemic control and functional properties without any negative side effects [41, 150]. Besides, RSV-induced cardioprotection in the hearts of diabetic models was also shown to be time dependent due to its low bioavailability and rapid metabolism [41]. Bresciani et al. found that RSV started to bioaccumulate in myocardial tissue after the third week in diabetic rats i.p. injected with 1 and 5 mg/kg/day of RSV and showed myocardial dose-concentration differences after 6 weeks of treatment [41]. Similarly, the cardioprotective effects were also shown to be induced in the period of 3-6 weeks of treatment and further increased over time in the present study. Taken together, a high-RSV dose accompanied by long-term treatment was suggested to maximize cardioprotection, while low-dose or short-term treatment exerted no protective effect against DCM. This result provides a basic requirement for future clinical trials designations.

## 6. Conclusion

According to current studies, both in vivo and in vitro, we presented the roles of RSV in the development of DCM. RSV serves as an upstream modulator of various molecular signaling pathways, such as Sirt1/3, AMPK, Akt, and MAPK pathways against DCM. Altered molecular signaling exerts cardioprotective effects, including antioxidative stress, anti-inflammatory, antiapoptotic, and proautophagic, which attenuate cardiac hypertrophy and eventually improve cardiac function in diabetes. In addition to the molecular signaling mechanisms, combined therapy of RSV and stem cells also showed great therapeutic potential for treating DCM. However, the clinical potential of RSV for patients with DCM has not been elucidated thus far. Whether RSV could play a role against DCM in patients should be investigated. Well-designed early phase clinical trials concerning specific doses and duration of RSV prescription are warranted to better evaluate the therapeutic value of RSV in DCM.

## Figures and Tables

**Table 1:** Concentrations of resveratrol and its main hydrophilic metabolites in the plasma and heart in some in vivo studies.

Ref.	Object	Treatment	Blood resveratrol			Heart resveratrol		
			Glucuronide conjugate	Sulfate conjugate	Free	Glucuronide conjugate	Sulfate conjugate	Free
[40]	Mice	Orally, a single dose of 150 mg/kg	*30 min later:* 99.83–241.45; *60* min *later:* 60.89–98.12 (*μ*M)	*30 min later:* 7.50–20.74; *60 min later:* 0.51–1.96 (*μ*M)	*30 min later:* 4.76–54.54; *60 min later:* 0.63–5.10 (*μ*M)	*30 min later:* 8.60–12.94; *60 min later:* 3.40–10.06 (nmol/g)	*30 min later:* 2.14–4.8; *60 min later:* 0.6–2.06 (nmol/g)	*30 min later:* 2.26–25.46; *60 min later:* 0.36–11.94 (nmol/g)
		Orally, 40 mg/kg/day for 3 months	<0.25 *μ*M	<0.13 *μ*M	ND	ND	ND	1.1–3.6 nmol/g

[41]	Diabetic rat	i.p., 5 mg/kg/day for 6 weeks	—	—	—	<0.03 nmol/g	<0.1 nmol/g	ND

[42]	Rat	Orally, a single dose of 50 or 150 mg/kg	<2290, <3740 (ng/mL)	<2020, <8710 (ng/mL)	<76.7, <847 (ng/mL)	—	—	—
		Orally, 50 or 150 mg/kg/day for 14 days	<1590, <2620 (ng/mL);	<2770, <10500 (ng/mL);	<176, <494 (ng/mL)	—	—	—

[43]	Human	Orally, 150 mg/kg/day for 30 days	—	—	182.59 ± 30.33 ng/mL	—	—	—

[44]	Human	Orally, 25 mg or 50 mg or 100 mg or 150 mg, 6 times/day for 2 days	—	—	24.3 ± 1.1, 23.9 ± 4.8, 27.6 ± 8.4, 23.5 ± 2.7 (ng/mL)	—	—	—

Abbreviation: ND: not detected; Ref.: reference.

**Table 2:** Studies revealing the cardioprotective effects of resveratrol against DCM development.

Model	Diabetic protocol	RSV treatment	Cardioprotective effect	Function recovery	Mechanisms	Ref.
SD rats/H9c2 cell	*T2DM* *In vivo*: HF diet+STZ i.p. injection (35 mg/kg) *In vitro*: HG culture (30 mmol/L)	*In vivo*: 50 mg/kg/d *In vitro*: 20 *μ*mol/L	Mitochondrial function **↑** Oxidative stress ↓ Glucose level ↓	NA	RSV activates SIRT1, thereby mediating PGC-1*α* deacetylation, improving mitochondrial function, and alleviating DCM injury. Besides, SIRT1 induces SOD activation, which contributes to the alleviation of oxidative stress in a DCM heart.	[25]

WT mice/H9c2/SIRT1 KO mice/SIRT1 KD H9c2	*T1DM* *In vivo*: STZ i.p. injection (150 mg/kg) *In vitro*: HG culture (33 mmol/L)	*In vivo*: 25 mg/kg/d∗5d *In vitro*: 50 *μ*mol/L∗48 h	Mitochondrial function **↑**	EF% **↑** FS% **↑**	RSV-induced SIRT1 activation ameliorates cardiac injuries in DCM through PGC-1*α*-mediated mitochondrial regulation.	[23]

SD rats/H9c2 cell	*T2DM* *In vivo*: HF diet *In vitro*: HG culture (33 mmol/L)	*In vivo*: 10 mg/kg/d∗8w *In vitro*: 30 *μ*mol/L∗1 h	Cardiac hypertrophy ↓ Oxidative stress ↓	NA	RSV-induced SIRT1 leads to the reduction of NF-*κ*B-p65 binding activity to DNA and attenuates cardiac hypertrophy and oxidative stress through reducing NADPH transcription.	[24]

SD rats/H9c2 cell	*T2DM* *In vivo*: HF diet+STZ i.p. injection (50 mg/kg) *In vitro*: AGEs (400 *μ*g/mL)	*In vivo*: NA *In vitro*: 10 *μ*mol/L∗2 h	Cardiomyocyte apoptosis ↓	NA	RSV activates SIRT1 and attenuates ER stress-induced cardiomyocyte apoptosis via PERK/eIF2*α*-, ATF6/CHOP-, and IRE1*α*/JNK-mediated pathways.	[80]

Adult CD1 mouse/neonatal rat cardiomyocytes	*T1DM* *In vivo*: STZ i.p. injection (150 mg/kg) *In vitro*: HG culture (33 mmol/L)	*In vivo*: 100 mg/kg/d∗2w *In vitro*: 5 *μ*mol/L	Cardiomyocyte apoptosis ↓ Cardiac fibrosis ↓ Ca^2+^ overload ↓	STD ↑ DTD ↑ FS ↑	RSV enhances SERCA2a expression and augments cardiomyocyte Ca^2+^ homeostasis by activating SIRT1, thereby inducing cardioprotection in DCM heart.	[66]

WT rats/H9c2 cell	*T1DM* *In vivo*: STZ i.p. injection (50 mg/kg) *In vitro*: HG culture	*In vivo*: 60 mg/kg/d∗12w *In vitro*: 25 *μ*mol/L∗12 h	Autophagic flux ↑ Cardiac fibrosis ↓	EF% ↑ FS% ↑ E/A ↑	RSV promotes cardiomyocyte autophagic flux via SIRT1/FOXO1/Rab7 axis, which subsequently attenuates cardiomyocyte apoptosis and oxidative stress injury in diabetic state.	[65]

SD rats/H9c2 cell	*T1DM* *In vivo*: STZ i.p. injection (50 mg/kg) *In vitro*: HG culture	*In vivo*: 25 mg/kg/d∗8w *In vitro*: NA	Mitochondrial function **↑** Cardiac fibrosis ↓	NA	RSV administration enhances SIRT3 expression, upregulates the acetylation status of TFAM, and improves the mitochondrial function.	[68]

Neonatal rat cardiomyocytes	HG culture (30 mmol/L)	50 *μ*mol/L∗24 h	Oxidative stress ↓ Cardiomyocyte apoptosis ↓	NA	RSV induces AMPK activation, thereby reducing ROS production and activating cardiac antioxidant enzyme activities.	[26]

H9c2 cell	HG culture (30 mmol/L)	25 *μ*mol/L∗24 h	Autophagic flux ↑ Cardiomyocyte apoptosis ↓	NA	RSV regulates the balance between autophagy and apoptotic machinery through activating AMPK, in conjunction with the following phosphorylation of the mTOR/p70S6K1/4EBP1 pathway and JNK1-mediated dissociation of the Beclin1-Bcl-2 complex.	[91]

SD rats/H9c2 cell	*T1DM* *In vivo*: STZ i.p. injection (65 mg/kg) *In vitro*: HG culture	*In vivo*: 25 mg/kg/d∗2w *In vitro*: 50 *μ*mol/L∗8 h	Glucose level ↓	NA	RSV activates AMPK/eNOS/NO, AMPK/Akt, Cav-1/eNOS pathways and Cav3 expression, thus augmenting Glut-4 translocation to cell surface and glucose uptake during hyperglycemia.	[67]

FVB mice	*T1DM* STZ i.p. injection (40 mg/kg)	10 mg/kg/d∗1 m	Oxidative stress ↓ Glucose level ↓ Cardiac fibrosis ↓	EF % ↑ FS % ↑ LVID ↓ (6 m) IVS ↓ (6 m) LVPW ↓ (6 m)	RSV prevents DCM by increasing Nrf2 expression and transcriptional activity.	[28]

Lepr^db^ mice/TNF KO mice	*T2DM* HF diet	20 mg/kg/d∗4w	Oxidative/nitrative stress ↓	LVEDV ↑ LVESV ↑ SV ↑	RSV suppresses TNF-*α*-induced NF-*κ*B activation and inhibits the expression and activation of NADPH oxidase and iNOS, which eventually attenuates oxidative/nitrative stress. Moreover, RSV also enhances eNOS expression in a NF-*κ*B-dependent manner, which contributes to diastolic relaxation.	[30]

SD rat	*T1DM* STZ i.p. injection (45 mg/kg)	80 mg/kg/d∗12w	Inflammation ↓ Glucose level ↓ Cardiac fibrosis ↓	LVSP ↑ LVEDP ↓ dp/dt_max_ ↑ dp/dt_min_ ↓ WHW/BW↓	RSV attenuates cardiac inflammatory response via downregulation of AT1R-ERK/p38MAPK signaling pathway in diabetic condition.	[69]

WT mice	*T1DM* STZ i.p. injection (40 mg/kg)	25 mg/kg/d∗2 m	Inflammation ↓ Glucose level ↓ Cardiac fibrosis ↓	NA	RSV prevents HMGB1/RAGE/TLR4/NF-*κ*B pathway, thereby reducing oxidative damage and inflammation in DCM hearts.	[29]

ZDF mice	*T2DM* Stock diet	200 mg/kg/d∗6w	Mitochondrial function **↑** Cardiac fibrosis ↓	LVDP ↑ dp/dt_max_ ↑ dp/dt_min_ ↓	RSV prevents P-CoA respiratory sensitivity, which decreases the accumulation of intracellular lipids, and alleviates mitochondrial dysfunction in ZDF mice heart.	[31]

SD rat/neonatal rat cardiomyocytes	*T1DM* *In vivo*: STZ i.p. injection (50 mg/kg) *In vitro*: HG culture (33 mmol/L)	*In vivo*: 10 mg/kg/d∗8w *In vitro*: 10 *μ*mol/L∗1 h	Cardiomyocyte apoptosis ↓ Glucose level ↓ Cardiac fibrosis ↓	LVEF ↑ LVFS ↑ LVIDd ↑ LVIDs ↑	RSV interrupts DCM development by inhibiting apoptosis via the PI3K/Akt/FoxO3a pathway.	[128]

SD rat	*T1DM* STZ i.p. injection (30 mg/kg)	10 mg/kg/d∗8w	Mitochondrial function **↑** Cardiomyocyte apoptosis ↓	EF% ↑ FS% ↑ LVSP ↑ dp/dt_max_ ↑ dp/dt_min_ ↓ LVEDd/v ↓ LVESd/v ↓	RSV treatment activates UCP2, thus improving cardiac function and inhibiting cardiomyocyte apoptosis, in conjunction with ameliorating mitochondrial function in diabetic rats.	[135]

C57BL/6 mice/mCFs	*T1DM* *In vivo*: STZ i.p. injection (40 mg/kg) *In vitro*: NA	*In vivo*: 25 mg/kg/d∗2 m *In vitro*: NA	Cardiac fibrosis ↓ Glucose level ↓	NA	RSV ameliorates fibrogenesis of DCM in STZ-induced diabetic mice by modulating ROS/ERK/TGF-*β*/periostin pathway	[137]

C57BL/6 mice/mCFs	*T1DM* *In vivo*: STZ i.p. injection (100 mg/kg) *In vitro*: HG culture (25 mmol/L)	*In vivo*: 10 mg/kg/d∗8w *In vitro*: 20 *μ*mol/L∗4 h	Cardiac fibrosis ↓	NA	RSV attenuates HG-induced collagen synthesis in CFs and also inhibits cardiac fibrosis in DCM heart by reducing the expression of the profibrogenic cytokine TGF-*β*1 and inhibiting TGF-*β*1–Smad3 signaling.	[131]

Wistar rats	*T2DM* STZ i.p. injection (50 mg/kg)+nicotinamide (100 mg/kg)	*In vivo*: 22.04 mg/kg/d∗6w	Cardiac fibrosis ↓	NA	RSV suppresses FGF2 and HSPGs expression, which alleviate cardiac fibrosis. Moreover, resveratrol also inhibits PPAR*γ*, which further attenuates cardiac fibrosis during high-glucose condition.	[140]

Abbreviation: SD rats: Sprague-Dawley rats; WT rats: wild-type rats; ZDF: Zucker diabetic fatty; KO: knockout; KD: knockdown; HF: high fat; i.p., intraperitoneal; EF%: ejection fraction; FS: fractional shortening; STD: systolic diameters; DTD: diastolic diameters; LVID: left ventricular end-diastolic diameter; IVS: interventricular septum; LVPW: LV posterior wall; LVEDV: LV end-diastolic volume; LVESV: LV end-systolic volume; SV: stroke volume; LVDP: left ventricular developed pressure; LVSP: left ventricular systolic pressure; dp/dt_max_ and dp/dt_min_: maximal first derivative of LV pressure increase and decrease, respectively; WHW/BW: the heart weight to body weight; LVEDP: LV end-diastolic pressure; LVEF: LV ejection fraction; LVFS: LV fractional shortening; LVIDd and LVIDs: LV internal dimensions at diastole and systole; LVEDd/v: LV end-diastolic dimension and volume; LVESd/v: LV end-systolic dimension; NA: not available; Ref.: reference.
